# Immunoregulatory biological response modifiers: effect of cytokines on septic shock

**DOI:** 10.1155/S0962935193000675

**Published:** 1993

**Authors:** Michael A. Chirigos, Claudio De Simone

**Affiliations:** 1 National Cancer Institute National Institute of Health Bethesda MD USA; 2 Infectious Diseases Department of Internal Medicine University of L'Aquila L'Aquila Italy; 3 4 Cold Spring Court Potomac MD 20854 USA

## Abstract

Whole bacteria or bacterial components or their extracts were employed to restore or augment the immune system. Beneficial effects were attained with these agents in treating various diseases. These agents were named biological response modifiers (BRMs) because they regulated certain cellular components of the immune system. The cellular regulation induced by these BRMs was found to be due to cytokines. The cytokines were shown to act directly on the various cellular components and to provide therapeutic benefit in various autoimmune and immune deficiency diseases. Overproduction of specific cytokines however leads to a deleterious effect on the host. Overproduction of tumour necrosis factor (endotoxin, lipopolysaccharide) leads to septic shock. Bacteraemia is the leading cause of overproduction of tumour necrosis factor (TNF). Septic shock in many cases leads to death. Several monoclonal antibodies to lipopolysaccharide (LPS) and anticytokines have demonstrated protection against septic shock.


Mediators of Inflammation 2, S17–S20 (1993)

### Immunological responses in patients with tuberculosis and *in vivo* effects of acetyl-L-carnitine oral administration

Emilio Jirillo,[1,CA] Maria Altamura,[1] Carlo Marcuccio,[1] Cosimo Tortorella,[2] Claudio De Simone[3] and Salvatore Antonaci[2]

[1]Immunologia and [2]Medicina Interna, Medical School, University of Bari, Bari, Italy; [3]Malattie Infettive, Medical School, University of L'Aquila degli Abruzzi, L'Aquila, Italy

[CA] Corresponding Author

**TUBERCULOSIS (TBC) is characterized by a complex immune response which parallels the clinical course of the disease. In this respect, acquired resistance, delayed hypersensitivity reaction and anergy are the main types of immune reactivity to mycobacterial antigens. In view of the presence of nonspecific and specific immune deficits in TBC patients, a clinical trial was carried out in a group of 20 individuals with active pulmonary TBC by oral administration of acetyl-L-carnitine (ALC). This drug, which has been shown to possess immunomodulating activities, was able to upregulate the T-dependent antibacterial activity in TBC patients after 30 days' treatment, while the same activity decreased in patients receiving placebo only. On the other hand, ALC did not modify serum levels of tumour necrosis factor-α, in the same individuals. This cytokine plays a detrimental rather than beneficial role in TBC pathogenesis. In the light of these data, ALC seems to be a powerful immunomodulator in the course of *Mycobacterium tuberculosis* infection and other mycobacteriosis.**

**Key words:** Acetyl-L-carnitine, Immune response, Lymphocytes, Macrophages, Tuberculosis, Tumour necrosis factor-α

### Introduction

Tuberculosis (TBC) is a pathological process characterized by a broad spectrum of immunological responses which correlate with the clinical course of the disease.[1–3] In TBC, immune responsiveness depends on three basic mechanisms: (1) acquired cellular resistance; (2) delayed type hypersensitivity (DTH); and (3) anergy.[1–3]

Acquired cellular resistance is a protective mechanism in which specific T-cells are activated by macrophages, the so-called antigen presenting cells.[4] With special reference to mycobacterial antigens, mainly proteins, e.g. heat shock proteins (HSP), are presented by macrophages to both human and murine $\gamma\delta^+$ cells.[5–8] On the other hand, murine $CD4^+$ and $CD8^+$ cells seem to participate to acquired resistance,[9] even if others claim that only $CD4^+$ cells are the major protective subset in long-term protective studies.[10] In humans, recent studies have pointed out that $CD4^+$ cells kill monocytes which contain mycobacteria regardless of the T-helper $(h)_1$–$Th_2$ subset distinction and of the HSP recognition.[11]

DTH is a detrimental reaction dependent on the sensitization of specific lymphocytes by mycobacterial antigens and usually leads to granuloma formation.[1–3] Granuloma, which consists of an accumulation of macrophages, may undergo either a protective evolution with microorganism neutralization or a caseification with tissue damage.[1–3] In the phase of macrophage accumulation, two inflammatory cytokines (CKs), namely the monocyte chemotactic protein-1 (MCP-1) and the macrophage inhibiting factor play an important role.[1–3] In the phase of tubercular granuloma evolution interferon-γ (IFN-γ) and interleukin-2 (IL-2) are protective,[12,13] while tumour necrosis factor-α (TNFα) is mostly involved in granuloma destruction.[14]

With special reference to TNFα, it has been reported that lipoarabinomannan (LAM) from the *M. tuberculosis* cell wall is responsible for the production of this CK,[15] as is also confirmed by studies on LAM-induced transcription of mRNA for several CKs, even including TNFα.[16] Furthermore, by analogy with other intracellular infections (e.g. *M. avium* and *M. bovis* infections, listerosis, toxoplasmosis and trypanosomiasis)[17] TNFα should also play a protective role in the case of *M. tuberculosis* infection. However, according to Filley and Rook[18] virulent TBC strains produce a factor which alters the normal function of TNFα, rendering it toxic to host tissue. Therefore, many clinical and laboratory findings in TBC patients may be ascribed to TNFα release, such as fever, weight loss, necrosis, intravascular coagulation and acute phase protein release.[19] On the other hand, data of Barnes *et al.*[20] support a protective role for TNFα in the course of pleuritis, which usually resolves in the absence of therapy. Moreover, Takashima *et al.*[21] report a reduction of TNFα in patients with chronic

© 1993 Rapid Communications of Oxford Ltd

TBC. Taken together, these findings attribute a noxious role to TNFα in the course of TBC, while beneficial effects of this CK may arise when it is moderately produced or locally released as in the case of pleural effusion.

Anergy is a clinical condition of depressed immune function, as observed in patients with advanced refractory TBC.[1-3] Several mechanisms have been postulated for explaining the immunosuppression in TBC patients. In this respect, bacterial cell wall components, e.g. D-arabino-D-galactan, can downregulate Fc receptor (R) function on cell membranes by circulating in the form of immunocomplexes.[22] Furthermore, prostaglandin $E_2$ release by monocytes may reduce IL-2 production and decrease expression of IL-2 R.[23] Another immunosuppressive agent is represented by IL-1, which inhibits T-lymphocyte proliferation by protein purified derivative (PPD) when produced in exaggerated amounts.[24] Finally, a 25 kDa glycoprotein derived from *M. tuberculosis* has been shown to inhibit phagocytic functions.[25]

These data emphasize that a plethora of suppressive factors may be involved in the depression of immune response in TBC patients and their recognition is important for the establishment of therapeutic strategies.

### Monitoring of the Immune Status in Patients with TBC

Until now, many studies have been focused on T-lymphocyte functions in TBC individuals and, in particular, T-cell response to mitogens and antigens has been evaluated. In contrast, less information is available on polymorphonuclear cell (PMN) and monocyte functions, and on B-cell activity.

In a recent study, a group of 14 patients with TBC (seven individuals with acute disease and seven individuals with chronic disease) have been evaluated in terms of phagocytic and B-cell functions.[26] PMN and monocyte activities were assessed by determining their chemotactic capacity and ability to phagocytose *Candida albicans.* At the same time, the release of two inflammatory CKs, leucocyte inhibiting factor (LIF) and MCP-1 was determined Results show that chemotaxis, phagocytosis and killing were reduced regardless of the disease status.[26] The impaired release of LIF and MCP-1 could also explain the altered phagocytic activity.

Assessment of B-cell responsiveness was performed by evaluating *in vitro* antibody synthesis in a plaque-forming cell system, using pokeweed mitogen (PWM) as a polyclonal activator and PPD as a specific antigen. In patients with acute TBC, anti-PPD antibody response was significantly augmented, while in patients with acute and chronic TBC PWM-induced antibody production fell within normal values.[26] These last results are in accordance with findings of others who detected elevated IgG serum levels to PPD in acute TBC.[27] Moreover, a strict correlation was found between ELISA positivity for anti-PPD IgG antibody and TBC diagnosis.[28] In chronic patients the lack of anti-PPD antibody response may depend on B-cell immunosuppression, despite an elevated frequency of B-cells.

The bulk of the above data support the usefulness of monitoring different compartments of the immune system in TBC patients, since deficits in these individuals seem to be more vast than expected.

### Oral Administration of Acetyl-L-carnitine in Patients with Active Pulmonary Tuberculosis

Much evidence supports the concept that the immune response plays an important role in the pathogenesis of TBC. Quite interestingly, the authors' previous results emphasize that even patients in the acute phase exhibit multiple deficits of either nonspecific or specific immunity. Now, the question arises concerning the opportunity to immunomodulate these subjects in order to avoid superimposition of other infections (e.g. historically, measles; nowadays, HIV infection). However, the risk exists that classical biological response modifiers (BRM), which enhance immune response via CK release,[29] may trigger detrimental DHRs for the host. In this framework, it was reasoned that a putative BRM, which may increase the metabolic performance of immune cells rather than inducing release of noxious mediators, should be more appropriate for treating TBC subjects.

Just recently, ALC, a substance which is actively involved in either the transport and oxidation of fatty acids into mitochondria, or in the production and incorporation of their unsaturated forms into membrane phospholipids,[30] has been shown to possess immunoregulatory properties. Studies in this direction have, in fact, demonstrated, that ALC reduced macrophage and lymphocyte function decline in aged rats[31] and enhanced mitogen-induced lymphocyte proliferation in the elderly.[32] In AIDS subjects treated with ALC an improvement of immunocompetence was found[33] and this correlates with the reduced levels of carnitine detected in these patients.[34]

On this basis, 20 subjects with active pulmonary TBC were enrolled in a double blind, randomized, controlled parallel study.[35] Ten patients were treated orally with 2 g ALC/day (Nicetile, Sigma-Tau, Pomezia, Italy) for 30 days. The control group received placebo only. Two main immune parameters were followed up at day 0 and day 30, namely the antibacterial activity and TNFα serum levels.

Antibacterial activity is a T-cell function dependent on $CD4^+$ and $CD8^+$ cell activity which represents a good index of the host resistance against pathogens, even including *M. tuberculosis*.[36] This activity was depressed in both groups of patients at time 0, but, while it increased in the ALC-treated individuals, it decreased in the subjects receiving placebo only. Several hypotheses can be formulated to explain the immunomodulating activities of ALC: (1) ALC may act by supplementation of energy to lymphocytes via ATP; (2) chemotherapy may impair lymphocytic functions in these patients[37,38] and ALC eventually potentiates the reduced antibacterial activity and/or prevents iatrogenic immunosuppression; or (3) ALC can modulate the hypothalamus–pituitary–adrenal axis with release of enhancing neurohormones and neuropeptides.[39]

As far as determination of serum levels of TNFα is concerned, no differences were seen between the two groups, since mean values of this CK fluctuated within normal ranges before and after treatment. However, in three cases within the ALC-treated group and in two cases within the placebo group TNFα levels increased by day 30. Although anamnesis, clinical examination, disease and state and other related blood parameters were taken into consideration, a reasonable explanation for understanding the above finding was not achieved. It is noteworthy that this present data confirms that reported by Rook *et al.*,[19] who also found normal serum levels of TNFα in TBC patients. In this context, to extend our data further, studies will evaluate *in vitro* TNFα release from LAM-stimulated monocytes in ALC-treated TBC patients. In fact, detection of TNFα in serum can be prevented by the presence of inhibitors found in TBC and sarcoidosis.[40]

### Therapeutic Application of Acetyl-L-carnitine in Mycobacteriosis

Tuberculosis and leprosy are the two major mycobacterial infections in which a deficit of Th cells has been recognized, mostly in terms of release of protective CKs, such as IL-2 and IFN-γ.[1 3,12,13,41 43] On the other hand, according to Grau *et al.*[44] TNFα may cause both protection and clinical manifestations in mycobacterial disease and pathology should be interpreted as the consequence of the protective mechanism. Therefore, a pharmacological modulation of the immune response in mycobacteriosis should be applied to the T-cell compartment, excluding the monocyte–macrophage system whose products, e.g. IL-1 and TNFα, are more detrimental than beneficial.[24,44]

The authors' data indicate that ALC may represent a suitable BRM for treating mycobacteriosis, since it increases T-cell dependent antibacterial activity without effect on *in vivo* TNFα production in the affected patients. In this respect, the improvement of clinical and immunological findings observed in ALC-treated AIDS patients[33] indirectly confirms that this compound acts on lymphocytes but not on cellular sources of TNFα. In fact, several papers support the prominent role of TNFα as the cofactor in the triggering and worsening of HIV infection.[45 48]

Finally, even if these preliminary results seem to support the immunomodulating capacities of ALC, long-term studies and monitoring of additional immune parameters are needed on a broader sample of the population affected by mycobacterial disease.

### References

1. Daniel TM, Oxtoby MJ, Pinto E, *et al.* The immune spectrum in patients with pulmonary tuberculosis. *Am Rev Respir Dis* 1981; **123**: 556 569.

2. Dannenberg AM. Immune mechanisms in the pathogenesis of pulmonary tuberculosis. *Rev Infect Dis* 1989; **11 (Suppl)**: S369 S378.

3. Jirillo E, Munno I, Tortorella C, *et al.* The immune response to mycobacterial infection. *Med Sci Res* 1989; **17**: 929 931.

4. Ellner JJ, Spagnuolo PJ, Schacter BZ. Augmentation of selective monocyte functions in tuberculosis. *J Infect Dis* 1981; **144**: 391 397.

5. Lamb JR, Lathigra R, Rothbard JB, *et al.* Identification of mycobacterial antigens recognized by T lymphocytes. *Rev Infect Dis* 1989; **11 (Suppl)**: S443 S447.

6. Munk ME, Schoel B, Modrow, S, *et al.* T lymphocytes from healthy individuals with specificity to self-epitopes shared by the mycobacterial and human 65-kilodalton heat shock protein. *J Immunol* 1989; **143**: 2844 2849.

7. Kabelitz D, Bender A, Schondelmaier S, *et al.* A large fraction of human peripheral blood $\gamma/\delta^+$T-cells is activated by *Mycobacterium tuberculosis* but not by its 65-kDa heat shock protein. *J Exp Med* 1990; **171**: 667 669.

8. O'Brien RL, Happ MP, Dallas A, *et al.* Stimulation of a major subset of lymphocyte-expressing T-cell receptor γδ by an antigen derived from *Mycobacterium tuberculosis*. *Cell* 1989; **57**: 667 674.

9. Orme IM, Miller ES, Roberts AD, *et al.* T-lymphocytes mediating protection and cellular cytolysis during the couse of *Mycobacterium tuberculosis* infection. Evidence for different kinetics and recognition of a wide spectrum of protein antigens. *J Immunol* 1992; **148**: 189 196.

10. Leveton C, Barnass S, Champion B, *et al.* T-cell mediated protection of mice against virulent *Mycobacterium tuberculosis*. *Infect Immun* 1989; **57**: 390 395.

11. Boom WH, Wallis RS, Chevernak KA. Human *Mycobacterium tuberculosis*-reactive $CD4^+$ T-cell clones; heterogeneity in antigen recognition, cytokine production, and cytotoxicity for mononuclear phagocytes. *Infect Immun* 1991; **59**: 2737 2743.

12. Toossi R, Kleinhenz M, Ellner JJ. Defective interleukin-2 production and responsiveness in human pulmonary tuberculosis. *J Exp Med* 1986; **163**: 1162 1172.

13. Onwubalili JK, Scott GM, Robinson JA. Deficient immune interferon production in tuberculosis. *Clin Exp Immunol* 1985; **59**: 405 410.

14. Kunkel SL, Chensue SW, Streiter RM, *et al.* Cellular and molecular aspects of granulomatous inflammation. *Am J Respir Cell Mol Biol* 1988; **1**: 439 447.

15. Moreno C, Taverne J, Mehlert A, *et al.* Lipoarabinomannan from *Mycobacterium tuberculosis* induces the production of tumour necrosis factor from human and murine macrophages. *Clin Exp Immunol* 1989; **76**: 240 245.

16. Barnes PF, Chatterjee D, Abrams JS, *et al.* Cytokine production induced by *Mycobacterium tuberculosis* lipoarabinomannan. Relationship to chemical structure. *J Immunol* 1992; **149**: 541 547.

17. Fong Y, Lowry SF. Tumour necrosis factor in the pathophysiology of infection and sepsis. *Clin Immunol Immunopathol* 1990; **55**: 157 162.

18. Filley EA, Rook GAW. Effect of mycobacteria on sensitivity to the cytotoxic effects of tumour necrosis factor. *Infect Immun* 1991; **59**: 2567 2572.

19. Rook GAW, Attiyah RA, Foley N. The role of cytokines in the immunopathology of tuberculosis and the regulation of agalactosyl IgG. *Lymphokine Res* 1989; **8**: 323 328.

20. Barnes PF, Fong SJ, Brennan PJ, *et al.* Local production of tumour necrosis factor and IFN-γ in tuberculous pleuritis. *J Immunol* 1990; **145**: 149 154.

21. Takashima T, Ueta C, Tsuyuguchi I, *et al.* Production of tumour necrosis factor alpha by monocytes from patients with pulmonary tuberculosis. *Infect Immun* 1990; **58**: 3286 3292.

22. Kleinhenz ME, Ellner JJ, Spagnuolo P, *et al.* Suppression of lymphocyte responses by tuberculous plasma and mycobacterial arabinogalactan: monocyte dependence and indomethacin reversibility. *J Clin Invest* 1981; **68**: 153–162.
23. Ellner JJ, Wallis RS. Immunologic aspects of mycobacterial infections. *Rev Infect Dis* 1989; **2 (Suppl 2)**: S455 S459.
24. Fujiwara H, Kleinhenz ME, Wallis R, *et al.* Increased interleukin-1 production and monocyte suppressor cell activity associated with human tuberculosis. *Am Rev Respir Dis* 1986; **133**: 73 77.
25. Wadee AA, Clara AM. A 25-kilodalton fraction from *Mycobacterium tuberculosis* that inhibits hexose monophosphate shunt activity, lysozyme release, and $H_2O_2$ production: reversal by gamma interferon. *Infect Immun* 1989; **57**: 864 869.
26. Antonaci S, Jirillo E, Polignano A, *et al.* Evaluation of phagocyte functions, inflammatory lymphokine activities and *in vitro* antibody synthesis in patients with active and chronic pulmonary tuberculosis. *Cytobios* 1991; **67**: 135 144.
27. Radin RC, Zeiss CR, Phair JP. Antibodies to purified protein derivative in different immunoglobulins classes in the diagnosis of tuberculosis in man. *Int Arch Allergy Appl Immunol* 1983; **70**: 25 29.
28. Daniel TM, Debanne SM. The serodiagnosis of tuberculosis and other mycobacterial diseases by enzyme-linked immunosorbent assay. *Am Rev Respir Dis* 1987; **135**: 1137–1151.
29. Antonaci S, Tortorella C, Jirillo E. Immunomodulating properties of thymic hormones in immunocompromised host. *Med Sci Res* 1991; **19**: 539 541.
30. Bremer J. Carnitine. Metabolism and functions. *Physiol Rev* 1983; **63**: 1420 1480.
31. Foresta P, Albertoni C, Ramacci MT *et al.* Immunological parameters in aged rats: effects of acetyl-L-carnitine. In: De Simone C, Martelli EA, eds. *Stress, immunity and ageing. A role for acetyl-L-carnitine.* Amsterdam: Elsevier Science Publishers B.V. (Biomedical Division), 1989; 97 107.
32. Monti D, Cossarizza A, Troiano L *et al.* Immunomodulatory properties of L-acetyl-carnitine on lymphocytes from young and old humans. In: De Simone C, Martelli EA, eds. *Stress, immunity and ageing. A role for acetyl-L-carnitine.* Amsterdam: Elsevier Science Publishers B.V. (Biomedical Division), 1989; 183 196.
33. De Simone C, Calvani M, Catania S, *et al.* Acetyl-L-carnitine as a modulator of the neuroendocrine-immune interaction in $HIV^+$ subjects. In: De Simone C, Martelli EA, eds. *Stress, immunity and ageing. A role for acetyl-L-carnitine.* Amsterdam: Elsevier Science Publishers B.V. (Biomedical Division), 1989; 125 138.
34. De Simone C, Tzantzoglou S, Jirillo E, *et al.* L-Carnitine deficiency in AIDS patients. *AIDS* 1992; **6**: 203 205.
35. Jirillo E, Altamura M, Munno I, *et al.* Effects of acetyl-L-carnitine oral administration on lymphocyte antibacterial activity and TNF-α levels in patients with active pulmonary tuberculosis. A randomized double blind versus placebo study. *Immunopharmacol Immunotoxicol* 1991; **13**: 135 146.
36. Antonaci S, Tortorella C, De Simone C, *et al.* Role of bacterial lipopolysaccharides in the development of natural antibacterial activity mediated by human peripheral blood T lymphocytes. *Intern Rev Immunol* 1990; **6**: 237 245.
37. Villa ML, Rappocciolo G, Piazza P, *et al.* The interference of antibodies with antigen specific antibody response in man. *Int J Immunopharmacol* 1986; **8**: 805 809.
38. Youmans AS, Youmans GP. Effect of metabolic inhibitors on the formation of antibody to sheep erythrocytes, on the development of delayed hypersensitivity, and on immune response to infection with *Mycobacterium tuberculosis* in mice. *Infect Immun* 1978; **19**: 211 216.
39. Butler LD, Layman NK, Riedl PE, *et al.* Neuroendocrine regulation of *in vivo* cytokine production and effects: I. *In vivo* regulatory networks involving the neuroendocrine system, interleukin-1 and tumour necrosis factor-alpha. *J Neuroimmunol* 1989; **24**: 143 153.
40. Foley N, Lambert C, McNicol M, *et al.* An inhibitor of the toxicity of tumour necrosis factor in patients with sarcoidosis, tuberculosis and Crohn's disease. *Clin Exp Immunol* 1990; **80**: 395 399.
41. Nogueira N, Kaplan G, Levy E, *et al.* Defective gamma-interferon production in leprosy. Reversal with antigen and interleukin-2. *J Exp Med* 1983; **158**: 2165 2170.
42. Mohagheghpour N, Gelbe R, Larrick JW *et al.* Defective cell-mediated immunity in leprosy: failure of T-cells from lepromatous leprosy patients to respond to *Mycobacterium leprae* is associated with expression of interleukin-2 receptor and is not reconstituted by interleukin-2. *J Immunol* 1985; **135**: 1443–1449.
43. Munno I, Pellegrino NM, Fumo G, *et al.* Studies on lymphokine production in lepromatous leprosy patients. *Cytobios* 1990; **62**: 141 147.
44. Grau GE, Parida SK, Pointaire P, *et al.* TNF and mycobacteria. In: Beutler B, ed. *Tumour necrosis factor: the molecules and their emerging role in medicine.* New York: Raven Press, 1992; 329 340.
45. Jirillo E, Greco B, Munno I, *et al.* Demonstration of an exaggerated serum release of tumour necrosis factor alpha and interleukin-1β in HIV-infected patients: a possible correlation with circulating levels of bacterial lipopolysaccharides. In: Nowotny A, Spitzer JJ, Ziegler EJ, eds. *Cellular and molecular aspects of endotoxin reaction.* Amsterdam: Elsevier Science Publishers (Biomedical Division), 1990: 529 535.
46. Matsuyama T, Kobayashi N, Yamamoto N. Cytokines and HIV infection: is AIDS a tumour necrosis factor disease? *AIDS* 1991; **5**: 1405 1417.
47. Dezube BJ, Pardee AB, Beckett LA, *et al.* Cytokine dysregulation in AIDS: *in vivo* overexpression of mRNA tumour necrosis factor-α and its correlation with that of the inflammatory cytokine GRO. *J Acq Immune Defic Syndrome* 1992; **5**: 1099 1104.
48. Mastroianni CM, Paoletti F, Valenti C, *et al.* Tumour necrosis factor (TNFα) and neurological disorders in HIV infection. *J Neurol Neurosurg Psychiat* 1992; **55**: 219 221.

ACKNOWLEDGEMENTS. This paper was supported in part by grants from M.U.R.S.T. (40% and 60%), Rome, Italy.
